# Activity-Tuning of Supported Co–Ni Nanocatalysts via Composition and Morphology for Hydrogen Storage in MgH_2_

**DOI:** 10.3389/fchem.2019.00937

**Published:** 2020-01-28

**Authors:** Xiaoli Ding, Hongfei Ding, Yun Song, Cuili Xiang, Yongtao Li, Qingan Zhang

**Affiliations:** ^1^School of Materials Science and Engineering, Anhui University of Technology, Ma'anshan, China; ^2^School of Materials Science and Engineering, Guilin University of Electronic Technology, Guilin, China; ^3^School of Innovation and Entrepreneurship, Wanjiang University of Technology, Ma'anshan, China; ^4^Department of Materials Science, Fudan University, Shanghai, China

**Keywords:** hydrogen storage, MgH_2_, composition, morphology, catalysis

## Abstract

Developing cheap metal nanocatalysts with controllable catalytic activity is one of the critical challenges for improving hydrogen storage in magnesium (Mg). Here, it is shown that the activity of graphene-anchored Co–Ni nanocatalysts can be regulated effectively by tuning their composition and morphology, which results in significantly improved hydrogen storage in Mg. The catalytic activity of supported Co–Ni nanocatalysts is demonstrated to be highly dependent on their morphology and composition. When Ni was partly substituted by Co, the shape of these nanocatalysts was changed from spherical to plate-like, thus corresponding to a decrease in activity. These alterations intrinsically result in enhanced hydrogen storage properties of MgH_2_, i.e., not only does it exhibit a decreased peak desorption temperature but also a positive change in the initial activation for sorption. The results obtained provide a deep understanding of the tuning of catalytic activity via composition and morphology and further provide insights into improving hydrogen storage in Mg-based materials.

## Introduction

Magnesium (Mg)-hydrides are considered promising hydrogen-storage materials due to their high theoretical hydrogen capacity of ~7.6 wt%, low-cost, and good reversibility (Cao et al., 2015; Liu X. et al., 2015; Ding et al., 2017; Møller et al., 2017; Schneemann et al., 2018), even as compared to other high H-content complex hydrides (Zheng et al., 2008; Li et al., 2011, 2014a; Liu D. M. et al., 2015). As solid-state hydrogen storage media, their application is still hampered by a large reaction enthalpy of ~76 kJ· mol^−1^ H_2_ for desorption (Si et al., 2018), high activation barrier, and slow hydrogen atom diffusion (Paskevicius et al., 2010), which result in higher desorption temperature and sluggish kinetics. Many strategies such as catalytic doping (Liu et al., 2019; Wang et al., 2019), nanosizing (Xia et al., 2015; Fang et al., 2019), alloying (Li et al., 2014b; Wei et al., 2018), and ionic substitution (Ouyang et al., 2014) have been proposed to overcome these challenges.

Catalytic doping was demonstrated to be an effective approach for making hydrogen desorption from MgH_2_ easier at relatively lower temperature (Shen et al., 2018; Wang et al., 2018). More importantly, unlike alloying, which introduces more non-absorbed elements (Ouyang et al., 2014), the content of doped catalysts is very limited and does not decrease the effective hydrogen storage capacity. In the last two decades, various catalysts such as transition metals (Humphries et al., 2013; German and Gebauer, 2016), oxides (Lin et al., 2014), carbon (Yao et al., 2007), fluorides (Kalantzopoulos et al., 2014), metallic glasses (El-Eskandarany, 2016), and 2D MXenes (Liu et al., 2016) have thus been developed. Recently, carbon-supported transition metals, especially Ni, were found to possess a superior catalysis effect for promoting hydrogen storage in MgH_2_ (Ma et al., 2018), most of which focused on the type, amounts, and particle size of catalysts (Shevlin and Guo, 2013). However, so far, the dependence of the hydrogen sorption properties of MgH_2_ on the morphology, composition, and activity of supported nanocatalysts has not been established. The science behind it remains unclear, particular in terms of their intrinsic mechanisms for enhancement. It is therefore important to attempt to promote hydrogen storage of MgH_2_ based on consideration of catalytic activity tuned by morphology and/or composition.

In this work, we realize the regulation of the activity of graphene-supported Co-Ni nanocatalysts by changing their morphology and compositions and further examine their catalytic effects on hydrogen storage in Mg. It is demonstrated that when Ni was partly substituted by Co, the shape of these nanocatalysts is shifted from spherical to plate-like and that the catalytic activity of these supported Co–Ni nanocatalysts depends strongly on their morphology and composition. The correlations among catalyst composition, shape, activity, and altered hydrogen sorption of Mg are also discussed. The results obtained will deepen the understanding of catalytic hydrogen storage and provide insights into the design of novel catalysts for property improvement of Mg-based materials.

## Experimental Section

### Preparation of Graphene-Supported Co–Ni Nanocatalysts and Their Doped MgH_2_ Composites

The starting chemicals of cobalt chloride (CoCl_2_·6H_2_O, purity 99.99%), nickel chloride (NiCl_2_·6H_2_O, purity 99.3%), and hexamethylenetetramine (C_6_H_12_N_4_, purity 99%) were purchased from Alfa Aesar and used without further purification. First, CoCl_2_·6H_2_O, NiCl_2_·6H_2_O, and hexamethylenetetramine were dissolved in 1,000 ml of deionized water at different weight ratios and then refluxed for 6 h under nitrogen protection with continuous magnetic stirring. The light-pink precipitate was recovered by filtration and washed with deionized water and anhydrous ethanol in turn. Finally, it was air-dried at room temperature. By changing the starting mixing ratio of CoCl_2_·6H_2_O and NiCl_2_·6H_2_O, different kinds of Co/Ni(OH)_*x*_ precursors can be obtained according to the above process.

The precursor and graphene were first mixed manually at an optimized weight ratio of 7:3, and then the mixed powders were ball-milled on a planetary ball mill at 300 rpm with a ball-to-sample weight ratio of 60:1 for 6 h under an argon atmosphere. The catalyst powders were then thermally annealed at ~800°C for 5 h. Subsequently, the catalyst obtained was used to dope MgH_2_ by mixing them at a weight ratio of 95:5 for 2 h under a 0.5 MPa hydrogen atmosphere by using a planetary mill at 400 rpm with a 20:1 ball-to-powder ratio and are denoted as Ni/Co@G-doped MgH_2_ composites.

### Characterization and Hydrogen Sorption Measurements

To examine the microstructural evolution, scanning electron microscopy (SEM) and transmission electron microscopy (TEM) observations were carried out on a Shimadzu SUPERSCAN SSX-550 scanning electron microscope and JEOL JEM-2100 F instrument, respectively. The sample preparation was dispersed in a dried THF solvent and was then spread on a copper grid-supported holey carbon film. The high-angle annular dark-field (HAADF) image was detected by an FEI Tecnai F20 analytical scanning transmission electron microscope (STEM) operated at a 200 kV accelerating voltage. The thermogravimetric behaviors of the as-prepared samples were examined using a Netzsch STA 409 PC system with a ramping rate of 10°C/min under a flowing Ar atmosphere. The sorption properties were measured using an automated Sieverts-type apparatus, which allowed for the accurate determination of the amount of evolved hydrogen (Si et al., 2018). Typically, an ~1 g sample was loaded into a stainless-steel autoclave and was evacuated. Rapid heating in the furnace to the desired temperature was accomplished by immersing the sample chamber in it, and then the desorption was performed against a back pressure of 10 Pa, and the absorption was performed against a pressure of 5 MPa. Multi-cycling of ab-/desorption was repeated for activation until a constant value of absorbed hydrogen was obtained. After activation, the samples were placed under a vacuum to completely remove the residual hydrogen, and then the isothermal sorption kinetics and pressure–composition (P–C) isotherms were measured at various temperatures. All sample handling was carried out in an Ar-filled glove box.

## Results and Discussion

**Figure 1:**
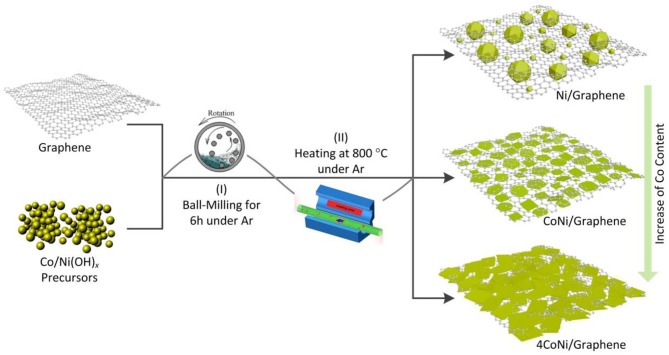
Schematic diagram of the preparation of Co-Ni nanocatalysts anchored to graphene by two steps. The morphology and size were changed significantly by adding Co into Ni catalysts.

Figure 1 shows a schematic diagram of the preparation of Co-Ni nanocatalysts anchored onto the graphene by two steps from the as-purchased graphene and Co/Ni(OH)_*x*_ precursors. These Co/Ni(OH)_*x*_ precursors, which were prepared by the hydrothermal method, exhibit small particle sizes and abundant active sites, which would facilitate the subsequent anchoring on graphene. Graphene and precursors with different mass ratios were first mixed by ball-milling and then heating at 800°C under an Ar atmosphere for carbon thermal reduction. By adjusting the composition of the Ni and Co ratio, different morphologies of particles and plates can be obtained for the Co-Ni metallic catalysts anchored on the graphene. Interestingly, Co incorporation does not alter the phase components of Co-Ni@G catalysts, suggesting its solid dissolution in the similar Ni lattice. With a further increase in Co content, the size of the Co-Ni based nanoplates can grow. Thus, we can realize control of the shape and size of the Co-Ni nanocatalysts prepared by this two-step method.

**Figure 2:**
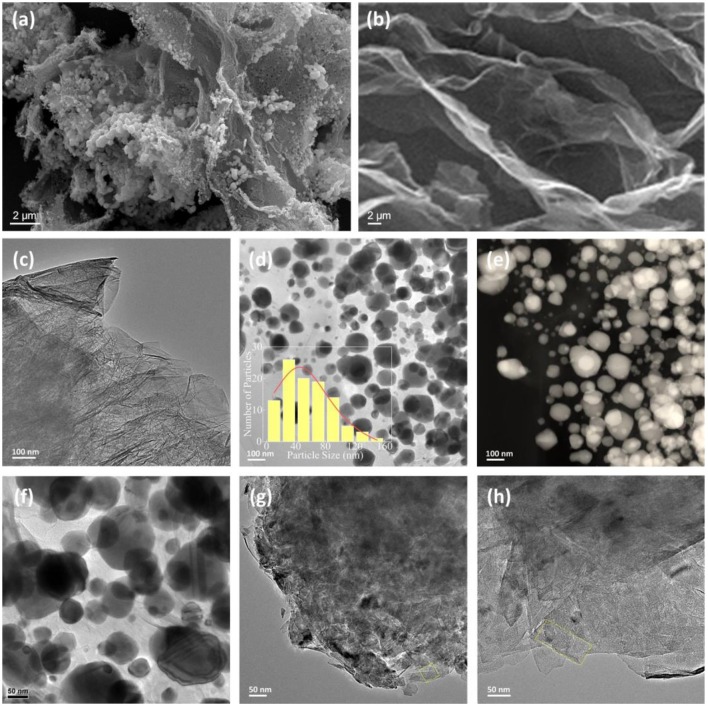
**(a)** SEM image of the Co/Ni(OH)_*x*_ precursor. **(b,c)** SEM and TEM images of as-purchased graphene. **(d,f)** TEM images and **(e)** HADDF-STEM image of the Ni nanoparticles anchored to graphene denoted as Ni@G, and the corresponding histogram for the particle size distribution (inset in **d**), showing an average diameter of 45 ± 7 nm. **(g,h)** TEM images of the CoNi nanoplates anchored to graphene denoted as CoNi@G and 4CoNi@G, respectively.

We further examine the morphological changes during the fabrication process to support the above schematic method. Figures 2a,b shows SEM images of the hydrothermal CoNi(OH)_*x*_ precursors and the as-prepared graphene. The precursors exhibit small particles with an aggregated structure, while the graphene shows an ultra-thin sheet structure somewhat like “corrugated paper,” as further confirmed by the TEM image shown in Figure 2c. Figure 2d shows the spherical morphology of the Ni@G with a broader size distribution from 10 to 160 nm, also confirmed by the HADDF-STEM image in Figure 2e. Further magnification of the image in Figure 2f shows that smaller particles of 10–30 nm along with bigger ones are observed in the Ni@G. When introducing Co into Ni catalysts, the shape of Ni catalysts changes from spherical to plate-like, as shown in Figure 2g. Interestingly, the plates sizes grow obviously with further increase in Co content, such as with change from CoNi to 4CoNi, as compared in Figures 2g,h. These graphene-supported Co-Ni nanocatalysts have different morphologies, both in shape and size, corresponding to changes in specific surface area and metallic active sites, which strongly suggests that they would exhibit distinct catalytic effects on the hydrogen storage in MgH_2_.

**Figure 3:**
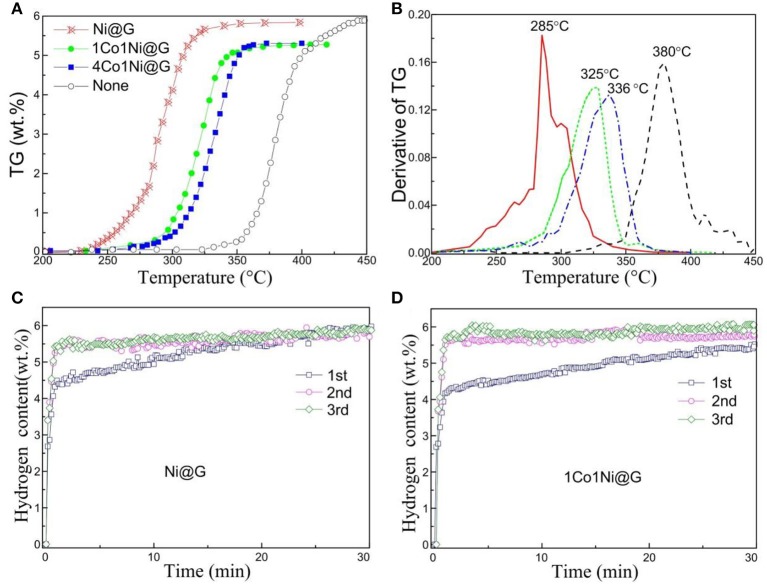
**(A)** TG curves and **(B)** derivative curves for MgH_2_ composites with no doping (“none”) and doped with Ni@G, CoNi@G, and 4CoNi@G catalysts. **(C,D)** Activation curves of the initial three desorption curves for typical Ni@G- and CoNi@G-doped MgH_2_ composites, respectively.

Figure 3A compares the TG curves of the non-doped and Ni@G-, CoNi@G-, and 4CoNi@G-doped MgH_2_ samples. As compared to pure MgH_2_, the hydrogen release from all Co-Ni@G catalyst-doped samples shifted to a lower temperature, where Ni@G shows the most significant improvement, i.e., the onset desorption temperature shifts from 325°C for MgH_2_ to 250°C and 225°C for CoNi@G-doped and Ni@G-doped MgH_2_, respectively. Similarly, the main peak for desorption shifts from 380°C for MgH_2_ to 336, 325, and 285°C for 4CoNi@G-, CoNi@G-, and Ni@G-doped MgH_2_, respectively, as shown in Figure 3B. These results strongly suggest that the Co-Ni nanocatalysts can cause a significant enhancement in desorption and that the introduction of Co deteriorates the catalytic effect of Ni. A similar phenomenon was also demonstrated by the activation testing at 300°C. As shown in Figures 3C,D, both Ni@G- and CoNi@G-doped MgH_2_ can be activated by two cycles. However, the initial desorption of CoNi@G-doped MgH_2_ shows relatively slow kinetics as compared to Ni@G-doped MgH_2_. The above results indicate that the catalytic effects of supported Co-Ni catalysts depend strongly on their composition and that the Ni element is more favorable than the Co element.

**Figure 4:**
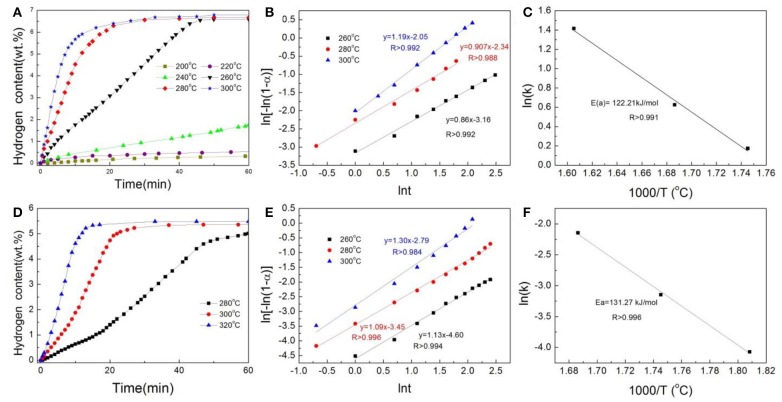
**(A)** Isothermal desorption curves, **(B)** Johnson-Mehl-Avrami (JMA) model plots of ln[–ln(1–α*(t)*)] vs. ln *t*, and **(C)** Arrhenius plot of the Ni@G-doped MgH_2_ at different temperatures. **(D)** Isothermal desorption curves, **(E)** Johnson-Mehl-Avrami (JMA) model plots of ln[–ln(1–α(t))] vs. lnt, and **(F)** Arrhenius plot of the CoNi@G-doped MgH_2_ at different temperatures.

Figures 4A,D shows the isothemal desorption curves of Ni@G- and CoNi@G-doped MgH_2_ samples. The Ni@G-doped MgH_2_ can release about 6.5 wt% H_2_ within 45 min at 260°C, while the desorption can be finished within 25 min at 280°C. In comparison, the CoNi@G-doped MgH_2_ sample exhibits relatively slow kinetics, with only 5 wt% H_2_ released within 60 min at 280°C. To quantitatively examine the distinct improvements in the desorption process, kinetic studies on the hydrogen release from the Ni@G-and CoNi@G-doped MgH_2_ were conducted by using the JMAK (*Johnson–Mehl–Avrami–Kolmogorov*) model (Li et al., 2014a). On the basis of the JMAK model, the desorption kinetics can be expressed by the following equation:

(1) $$\begin{array}{l} \ln\left[-\ln\left(1-\alpha \right)\right]=\eta \ln\;k+\eta \ln\;t \end{array}$$

where α is the reaction fraction, corresponding to the beginning and completion of the reaction, η is the Avrami exponent of reaction order, *k* is the rate constant, and *t* is time. For the experimental sample data, the plot of ln[–ln(1–α)] as a function of ln(*t*) was linear for each curve at the various temperatures, as shown in Figures 4B,E. After calculating the rate constant ln*k*, the activation energy *E*_a_ can be further determined by the Arrhenius equation:

(2) $$\begin{array}{l} k=k_{0}\exp\left(-E_{a}/RT\right) \end{array}$$

where *k*_0_ is the pre-exponential factor, *R* is the gas constant, and *T* is absolute temperature. From the slopes of the straight lines shown in Figures 4C,F, the activation energies *E*_a_ were calculated to be about 122 and 131 kJ mol^−1^ for the Ni@G- and CoNi@G-doped samples, both of which are lower than the reported 158 kJ mol^−1^ of pure MgH_2_. This clearly indicates that the activation energy can be significantly reduced by doping with Co-Ni nanocatalysts and that the element Ni is better than Co.

**Figure 5:**
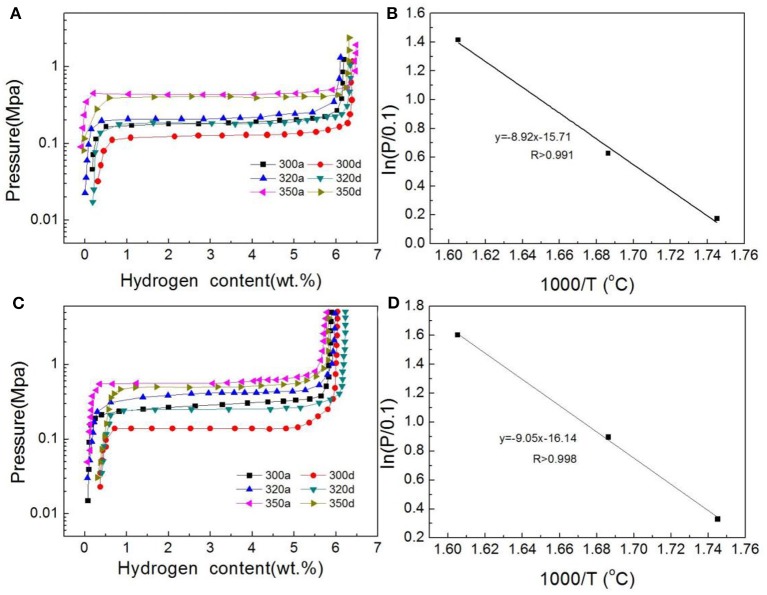
**(A)** P–C isotherms and **(B)** the van 't Hoff plots for the desorption plateaus of the Ni@G-doped MgH_2_ at different temperatures. **(C)** P–C isotherms and **(D)** the van 't Hoff plots for the desorption plateaus of the CoNi@G-doped MgH_2_ at different temperatures.

The pressure–composition isotherms for Ni@G- and CoNi@G-doped MgH_2_ at different temperatures are compared in Figures 5A,C. It can be seen that both Ni@G- and CoNi@G-doped MgH_2_ samples exhibit one flat plateau at different temperatures. Interestingly, CoNi@G-doped MgH_2_ shows a higher pressure of desorption plateaus than Ni@G-doped MgH_2_, suggesting its unfavorable thermodynamic properties. To evaluate these enthalpy changes for the two samples quantitatively, van ‘t Hoff plots of desorption based on their equilibrium pressures at various temperatures were drawn, as shown in Figures 5B,D. The obtained Δ*H*_d_ value of CoNi@G-doped MgH_2_ is about ~75 kJ mol^−1^ H_2_, higher than the Ni@G-doped sample, which has a value of ~74 kJ mol^−1^ H_2_, although both are lower than that of pure MgH_2_. These results clearly indicate that the desorption thermodynamics of MgH_2_ can be altered but that Ni is superior to Co for this purpose.

On the basis of the above results, we can conclude that the catalytic activity of the supported Co-Ni catalysts depends strongly on their composition and morphology, with Ni@G exhibiting the best improvement effects, which can be understood through the following two aspects: (i) the compositions are first of importance because of not only their different natural electronic structures but also their effects on the morphology of catalysts; (ii) the effects of small spherical catalysts are superior to those of nanoplates. This is because the small sphere morphology has a larger specific area, which provides more active sites and lower energy barriers for desorption, and it possesses abundant grain boundaries and defects that act as favorable channels for hydrogen diffusion. Furthermore, the introduction of Co indeed decreases the activity of Ni@G for hydrogen storage in MgH_2_, which can be understood through two aspects: i) introducing Co will increase of the particle size of the catalyst and its specific surface area, and thus its activity will be decreased, and ii) the electronegativity of Ni is larger than that of Co, corresponding to electron transport being easier in Ni atoms than in Co atoms, facilitating H atom dissociation and transfer. This explains why the introduction of Co exhibits an adverse catalytic effect for hydrogen storage.

## Conclusion

In conclusion, the catalytic activity of supported Co–Ni nanocatalysts is demonstrated to be highly dependent on their morphology and composition. When Ni was partly substituted by Co, the shape of these nanocatalysts changed from spheres to plates, thus corresponding to a decrease in their activity. These changes intrinsically not only result in a decreased peak desorption temperature but also positively change the initial activation for sorption, with a reduction in activation energy as compared with that of a pure sample. These results provide insights into the design of catalysts for improving the hydrogen sorption of Mg-based materials.

## Data Availability Statement

All datasets generated for this study are included in the article/supplementary material.

## Author Contributions

XD carried out the material preparation, analyzed the XRD, SEM, and TEM results, and co-wrote the paper. HD did the property test and wrote the paper. YS supervised all the experiments, especially the material preparation. CX discussed the results and the paper. YL and QZ attained the main financial support for the research, supervised all experiments, and revised the manuscript.

### Conflict of Interest

The authors declare that the research was conducted in the absence of any commercial or financial relationships that could be construed as a potential conflict of interest.
